# Long-term Weight Loss in a Primary Care–Anchored eHealth Lifestyle Coaching Program: Randomized Controlled Trial

**DOI:** 10.2196/39741

**Published:** 2022-09-23

**Authors:** Laura Hesseldal, Jeanette Reffstrup Christensen, Thomas Bastholm Olesen, Michael Hecht Olsen, Pernille Ravn Jakobsen, Ditte Hjorth Laursen, Jørgen Trankjær Lauridsen, Jesper Bo Nielsen, Jens Søndergaard, Carl Joakim Brandt

**Affiliations:** 1 Research Unit for General Practice Department of Public Health University of Southern Denmark Odense Denmark; 2 Steno Diabetes Center Zealand Holdbaek Denmark; 3 User Perspectives and Community-based Interventions Department of Public Health University of Southern Denmark Odense Denmark; 4 Research Unit of General Practice Aarhus University Aarhus Denmark; 5 Steno Diabetes Center Odense Odense University Hospital Odense Denmark; 6 Department of Internal Medicine Holbaek Hospital Holbaek Denmark; 7 Department of Regional Health Research University of Southern Denmark Odense Denmark; 8 Department of Public Health University of Copenhagen Copenhagen Denmark; 9 Department of Economics and Data Science University of Southern Denmark Odense Denmark

**Keywords:** obesity, digital behavioral coaching, health behavior change, interactive advice, lifestyle change, mobile intervention, patient engagement, telemedicine, eHealth, digital health, digital coach, weight loss, coaching, training, engagement, behavior changes, diabetes, type 2 diabetes, T2D

## Abstract

**Background:**

Long-term weight loss in people living with obesity can reduce the risk and progression of noncommunicable diseases. Observational studies suggest that digital coaching can lead to long-term weight loss.

**Objective:**

We investigated whether an eHealth lifestyle coaching program for people living with obesity with or without type 2 diabetes led to significant, long-term (12-month) weight loss compared to usual care.

**Methods:**

In a randomized controlled trial that took place in 50 municipalities in Denmark, 340 people living with obesity with or without type 2 diabetes were enrolled from April 16, 2018, to April 1, 2019, and randomized via an automated computer algorithm to an intervention (n=200) or a control (n=140) group. Patients were recruited via their general practitioners, the Danish diabetes organization, and social media. The digital coaching intervention consisted of an initial 1-hour face-to-face motivational interview followed by digital coaching using behavioral change techniques enabled by individual live monitoring. The primary outcome was change in body weight from baseline to 12 months.

**Results:**

Data were assessed for 200 participants, including 127 from the intervention group and 73 from the control group, who completed 12 months of follow-up. After 12 months, mean body weight and BMI were significantly reduced in both groups but significantly more so in the intervention group than the control group (–4.5 kg, 95% CI –5.6 to –3.4 vs –1.5 kg, 95% CI –2.7 to –0.2, respectively; *P*<.001; and –1.5 kg/m^2^, 95% CI –1.9 to –1.2 vs –0.5 kg/m^2^, 95% CI –0.9 to –0.1, respectively; *P*<.001). Hemoglobin A_1c_ was significantly reduced in both the intervention (–6.0 mmol/mol, 95% CI –7.7 to –4.3) and control (–4.9 mmol/mol, 95% CI –7.4 to –2.4) groups, without a significant group difference (all *P*>.46).

**Conclusions:**

Compared to usual care, digital lifestyle coaching can induce significant weight loss for people living with obesity, both with and without type 2 diabetes, after 12 months.

**Trial Registration:**

ClinicalTrials.gov NCT03788915; https://clinicaltrials.gov/ct2/show/NCT03788915

## Introduction

Long-term weight loss can reduce the risk, postpone the onset, and reduce the progression of noncommunicable diseases (NCDs) [1,2]. Numerous studies have shown that type 2 diabetes (T2D) can be slowed, halted, or even reversed through lifestyle changes, such as a low-calorie diet and increased physical activity [2,3]. This can lead to fewer long-term complications and probably a prolonged life expectancy [4]. Unfortunately, it is difficult for people living with obesity and T2D to achieve and maintain long-term weight loss [5]. Despite an intensive focus on T2D in general practice in Denmark, many patients are not treated optimally, nor do they follow recommendations for a healthy lifestyle [6]. Even though general practice is meant to support self-management and a healthy lifestyle, studies have shown that annual consultations seldom address lifestyle issues [7].

Meta-analyses and systematic reviews show that electronic health (eHealth) and mobile health (mHealth) solutions are significantly better than usual care, defined as routine diabetes self-care with no personalized feedback, at supporting weight loss in the short term (ie, within 3 to 6 months) for people living with obesity [8,9]. Behavior change techniques (BCTs) are an essential component of effective solutions. These involve automated, semi-automated, or human digital feedback [9,10]. Human feedback, particularly from health care professionals (HCPs), is most effective [11].

As described in detail in the study protocol [12], this study’s collaborative eHealth tool, called LIVA, has been developed based on the experiences of approximately 140,000 individuals who used the collaborative eHealth tool (version 1.0) over a period of 15 years [12]. Version 1.0 has been further developed into version 2.0 based on feedback from patients, general practitioners, and HCPs [13-15]. HCPs use the eHealth tool to conduct digital lifestyle coaching as a 1-hour-long, physical or virtual, face-to-face motivational interview. The participant and the HCP collaborate and agree on goals for relevant lifestyle activities, such as diet and exercise, that the patient is motivated to improve [12].

However, there is limited evidence on the potential for such solutions to lead to weight loss over the long term (ie, longer than 12 months) [8,9]. In this randomized controlled trial (RCT), we aimed to investigate whether digital coaching through a multifaceted eHealth tool could help people living with obesity, with or without T2D, to achieve and sustain more significant long-term weight loss than an equivalent control group receiving usual care.

## Methods

### Study Design and Ethical Approval

This study was part of an RCT that took place in 2 of the 5 regions in Denmark: the Capital Region of Denmark, with 28 municipalities, and the Region of Southern Denmark, with 22 municipalities. The study was carried out from April 2019 to October 2021. The study was approved by the scientific committee of the Region of Southern Denmark (S-20170183G). All methods are described in detail in the study protocol [12]. The study is registered at ClinicalTrials.gov (NCT03788915).

### Participants

In Denmark, lifestyle support is managed by local municipalities at health care centers. For this study, participants in municipal lifestyle programs within the participating regions were recruited through their local health care centers, general practitioners (GPs), the Danish diabetes organization, and social media. Participants who expressed a desire to participate could then register at the eHealth tool website [12]. After registration, participants were contacted by telephone by a research assistant, who ensured that the participant met the inclusion criteria for BMI (30-45 kg/m^2^) and age (18-70 years). The exclusion criteria were (1) a lack of internet access through a computer or smartphone, (2) pregnancy or planned pregnancy, and (3) presence of a serious or life-threatening disease, defined as a condition with less than a 1-year life expectancy.

### Randomization

Participants were randomized to the intervention group, who received usual care and the digital lifestyle coaching, or a control group, who received only the usual care preferred by the patient and their doctor. Randomization occurred after the participants had completed the medical examination via an automated computer algorithm in groups of 10 at a 6:4 ratio, where 60% of the recruited participants were randomized to the intervention group and the remaining 40% were assigned to the control group; this method was based on a pilot RCT [16] and is described in our protocol article [12]. Randomization was controlled to ensure that 50% of participants in both the intervention group and control group would be people living with obesity who had not previously been diagnosed with T2D, and to ensure that the other 50% of participants in both the intervention group and control group would be people living with obesity who had been diagnosed with T2D. Blinding the participants, the research assistant, and the health coach who provided the lifestyle coaching to all the participants who received the intervention was not possible after randomization. The research assistant and health coach had no role in analyzing or interpreting the data.

### Procedures

At the baseline meeting, the participants gave written informed consent and informed the research assistant about their use of medication. Afterwards, a brief medical examination was performed. The examination included measurements such as the participants’ height, measured in centimeters, without shoes; weight, measured with clothes but without shoes (we subtracted 1 kg for clothing); waist and hip circumference, measured with a tape measure around the waist, between the lower rib and pelvic curvature and hip, with one hand above the inguinal medial line (in keeping with the European Health Examination Survey guideline [17]); and blood pressure, measured in a seated position after 10 minutes of rest without speaking, using an electronic, automatic blood pressure monitor (Omron Model M3). Three blood pressure measurements were performed 1 minute apart, and the lowest measured value was recorded [18]. Hemoglobin A_1c_ (HbA_1c_), total cholesterol, high-density lipoprotein cholesterol (HDL-C), low-density lipoprotein cholesterol (LDL-C), and triglyceride (TG) levels were measured and assessed using finger-stick sampling with a device (Hemocue HbA_1c_ 501 Analyzer) that can measure HbA_1c_ in nonfasting blood samples [19]. To ensure the accuracy of the measurements, the Hemocue Analyzer was calibrated daily according to the manufacturer’s instructions. Additional calibration was done monthly using a special kit to test the sensitivity and specificity of the measurements. A strict protocol was followed for the collection of blood samples. This examination was performed at both 6 and 12 months.

All participants filled out the European Quality of Life-5 Dimensions (EQ-5D-5L) (an online questionnaire on sociodemographic characteristics) and the Short-Warwick-Edinburgh Mental Well-being Scale (SWEMWBS) [20,21]. The EQ-5D-5L descriptive system has 5 dimensions: mobility, self-care, usual activities, pain and discomfort, and anxiety and depression. Each dimension has 5 response levels: “no problems,” “slight problems,” “ moderate problems,” “severe problems,” and “unable to/extreme problems.” Responses are coded as single-digit numbers expressing the severity level selected for each dimension, which are then coded into a score ranging from 0.35 to 1.0. The SWEMWBS is a 7-item scale covering subjective well-being and psychological functioning. Each item is answered on a 5-point Likert scale, including “none of the time,” “rarely,” “some of the time,” “often,” and “all the time.” The summary index ranges from 7 to 35. Higher scores indicate higher well-being and psychological functioning [20,21]. The participants answered both questionnaires at baseline and after 6 and 12 months of follow-up.

### Intervention

After an initial 45-to-60-minute consultation with the HCP, the intervention group received the individualized digital lifestyle coaching and used the eHealth tool to complete daily records and to send remarks directly to the HCP. Based on individual goal setting created using the SMART (specific, measurable, attainable, relevant, timely) model [22], the health coach provided weekly asynchronous digital coaching for each participant that included inspiring them, commending them on goal attainment, and seeking to help them stay motivated [13]. The subsequent asynchronous eHealth coaching sessions were carried out once a week for the first 6 months and then once a month for the last 6 months, as maintenance. The eHealth tool application is further described in the Template of the Intervention Description and Replication (TIDieR) (Multimedia Appendices 1 and 2).

### Characteristics of HCPs

The HCPs who provided the digital lifestyle coaching through the eHealth tool were all educated as nurses, physiotherapists, dieticians, or occupational therapists. They all underwent special training in digital health coaching and had all practiced digital health coaching for at least 2 years. All participants were assigned a primary HCP so that there was a better chance of achieving a close and trusting professional relationship [12].

### Follow-up Procedure and COVID-19 Lockdown

**Figure 1:**
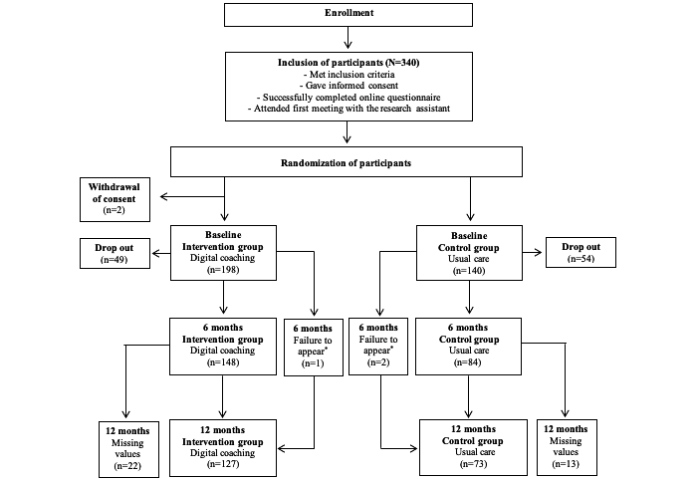
Flowchart of participation in 12-month randomized controlled trial. *Failed to appear at the assessment but appeared at the next follow-up.

After 6 and 12 months, the participants were invited to a brief medical examination, similar to the baseline examination, performed by a research assistant. To confirm patient-reported data, the same medical data were also retrieved from the shared medication record (abbreviated as “FMK” in Danish) and from laboratory results, measured at GP clinics. Participants were also asked to complete the same web-based questionnaire [12]. Participants were contacted 1 month before their 6- and 12-month assessments by telephone to schedule the assessment. If a participant did not respond, a voice mail was left explaining the purpose of the call. Another telephone call was made a week later and again 1 month later. Participants who had not responded to 4 different attempts were considered lost to follow-up. Due to the COVID-19 lockdown and national restrictions, some participants could not attend their 12-month assessment. Therefore, the 12-month assessment period was extended by 4 months, so that follow-up after baseline also covered 12 to 16 months. However, this extension might not have been sufficient to obtain 12-month follow-up data from all participants (Figure 1). Thus, this paper reports 12-month follow-up data from 126 participants in the intervention group and 71 participants in the control group who attended follow-up examinations at both 6 and 12 months.

### Outcomes

The primary outcome was reduction in mean body weight (BW), assessed as the difference between BW at baseline and at 12 months, and as the difference divided by baseline BW. The proportion of participants who had significant weight loss (ie, >5% of baseline BW [23]) was also assessed at 6 and 12 months. Our secondary outcome was the change in HbA_1c_ at 6 and 12 months compared to baseline. The tertiary outcomes were body composition (BMI and waist/hip ratio), lipid levels (total cholesterol, LDL-C, HDL-C, and TG), blood pressure (systolic and diastolic), and changes in mental health and quality of life. All differences in tertiary outcomes were calculated from baseline to 6 months and baseline to 12 months.

### Statistical Analysis

All analyses used Stata/BE (version 17.0; StataCorp) and were performed on data from participants who attended the 12-month follow-up; other participants were considered dropouts. Baseline characteristics of all participants allocated to the intervention and control groups were analyzed descriptively. The statistical significance of differences in baseline characteristics of the participants who attended the 12-month follow-up was assessed with the Student *t* test and the Kruskal-Wallis test. The statistical significance of between-group differences in outcomes at 6 and 12 months was assessed with either a 1-way ANOVA or the chi-square test. Statistical significance was set at 2-tailed *P*<.05. In addition, we performed a per protocol analysis by using only data from participants who had been using the eHealth tool for 365 days or more. Finally, we performed a regression analysis that included an interaction term to determine whether participants with T2D responded differently to the intervention.

## Results

### Participant Characteristics

From April 16, 2018, to April 1, 2019, 340 participants were randomized. Two participants in the intervention group decided to withdraw their consent; thus, a total of 338 participants were included. The intervention group included 198 participants (128/198 female, 65%) and the control group included 140 participants (85/140 female, 61%) (Figure 1). At baseline, the intervention and control groups were comparable (Table 1). Participants’ mean BW was 103.7 kg, their mean BMI was 35.3 kg/m^2^, and their mean HbA_1c_ was 6.6% (Table 1).

A total of 200 participants completed the 12-month follow-up (Figure 1). Participants who dropped out of the study (ie, did not complete the 12-month follow-up) were generally not different from the active participants. However, significantly fewer participants who dropped out were married, more were unmarried (including divorce), and they had slightly higher diastolic blood pressure and lower quality of life (Multimedia Appendix 3), although there were no significant differences within the intervention group or the control group. At baseline, there were significantly fewer participants receiving metformin, SGLT2, or calcium antagonists in the group of participants who dropped out (Multimedia Appendix 3).

**Table 1:** Baseline characteristics of participants.

Characteristics		Intervention group (n=127)	Control group (n=73)	Total (N=200)
Age (years), mean (SD)		52.3 (10)	52.3 (12)	52.3 (11)
**Sex, n (%)**				
	Female	86 (68)	41 (56)	127 (64)
	Male	41 (32)	32 (44)	73 (37)
**Diabetes, n (%)**				
	Yes	62 (50)	36 (49)	98 (49)
	No	65 (51)	37 (51)	102 (51)
**Education, n (%)**				
	None	19 (15)	15 (21)	34 (17)
	Short	33 (26)	19 (26)	52 (26)
	Medium	61 (48)	30 (41)	91 (46)
	Long	12 (9)	9 (12)	21 (11)
	Don’t know	2 (2)	0 (0)	2 (1)
**Marital status, n (%)**				
	Married	92 (72)	49 (67)	141 (71)
	Unmarried^a^	33 (26)	23 (32)	56 (28)
	Widowed	2 (2)	1 (1)	3 (2)
**Occupational status, n (%)**				
	Employed	96 (76)	48 (66)	144 (72)
	Out of work^b^	10 (8)	6 (8)	16 (8)
	Retired	20 (16)	17 (23)	37 (19)
	Student	1 (0)	2 (3)	3 (2)
**Body composition, mean (SD)**				
	Weight (kg)	103.0 (15.7)	104.9 (15.8)	103.7 (15.7)
	BMI (kg/m^2^)	34.8 (3.7)	36.0 (3.8)	35.3 (3.8)
	Hip circumference (cm)	121.1 (9.6)	121.7 (10.2)	121.3 (9.8)
	Waist circumference (cm)	117.7 (11.4)	121.2 (11.7)	119.0 (11.6)
	Waist to hip ratio	1.0 (0.1)	1.0 (0.1)	1.0 (0.1)
**Glycemic control**				
	HbA_1c_^c^ (%), mean (SD)	6.6 (1.3)	6.6 (1.3)	6.6 (1.3)
	HbA_1c_ (mmol), mean (SD)	48.3 (13.6)	48.4 (14.0)	48.3 (13.7)
	HbA_1c_ <6.5%, n (%)	70 (55)	41 (56)	111 (56)
**Blood pressure, mean (SD)**				
	Systolic (mm Hg)	130.6 (13.8)	131.4 (16.6)	130.9 (14.8)
	Diastolic (mm Hg)	86.0 (8.1)	86.5 (10.4)	86.2 (9.0)
**Lipids**				
	Total cholesterol (mmol/l), mean (SD)	4.9 (1.3)	4.8 (1.1)	4.9 (1.2)
	High density lipoprotein (mmol/l), median (IQR)	1.2 (0.7)	1.2 (0.6)	1.2 (0.5)
	Low density lipoprotein (mmol/l), median (IQR)	2.3 (1.4)	2.2 (1.6)	2.2 (1.5)
	Triglycerides (mmol/l), median (IQR)	2.6 (2.2)	2.7 (2.5)	2.6 (2.3)
Mental Health score^d^ , mean (SD)		24.9 (3.2)	24.5 (3.9)	24.8 (3.5)
Quality of life score^e^, mean (SD)		0.8 (0.1)	0.8 (0.1)	0.8 (0.1)

^a^Single or divorced.

^b^On maternity leave or receiving unemployment or cash benefits.

^c^HbA_1c_: hemoglobin A_1c_.

^d^Measured with Short-Warwick-Edinburgh Mental Well-being Scale; index ranges from 7-35.

^e^Index calculated based on the EQ-5D-5L; ranges from 0.35 to 1.0.

### Primary Outcome

At the 6-month follow-up, BW was significantly reduced in the intervention group (–4.5 kg, 95% CI –5.4 to –3.5) and not significantly reduced in the control group (–0.3 kg, 95% CI –1.1 to 0.4). This between-group difference was statistically significant (*P*<.001) (Table 2). Our primary outcome, BW at the 12-month follow-up, was significantly reduced in both the intervention group (–4.5 kg, 95% CI –5.6 to –3.4) and the control group (–1.5 kg, 95% CI –2.7 to –0.2); the reduction in the intervention group was significantly greater (*P*<.001) (Table 3). There was a significant weight loss (defined as >5% BW, *P*=.01) in a greater proportion of participants in the intervention group (48/127, 37.8%) than the control group (14/73,19%). The same pattern was seen among the per protocol participants (ie, the participants who used the eHealth tool for 365 days or more).

**Figure 2:**
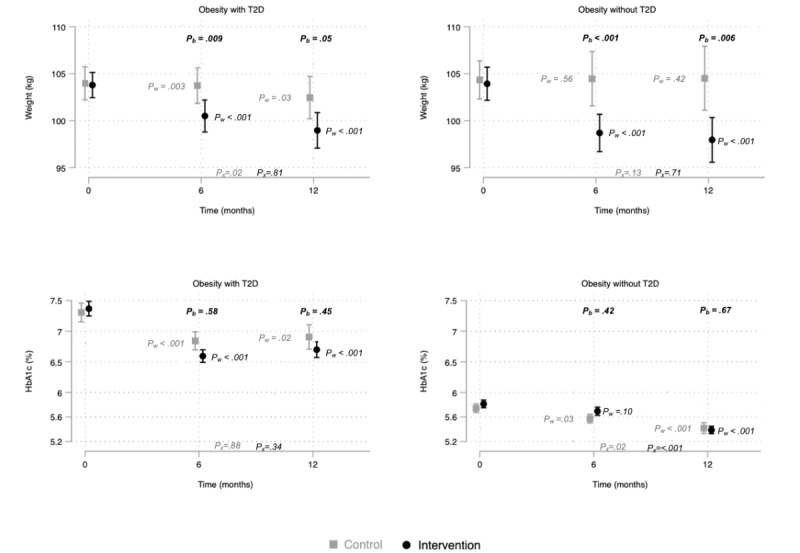
Body weight and hemoglobin A_1c_ at baseline, at the 6-month follow-up (n=197), and at the 12-month follow-up (n=200) in subgroups with and without type 2 diabetes. Dots indicate the mean and lines indicate the standard error of the mean. P_w_: *P* value for changes from baseline within groups; P_b_: *P* value for changes from baseline between groups; P_x_: *P* value for changes from 6 to 12 months within groups; HbA_1c_: hemoglobin A_1c_; T2D: type 2 diabetes.

Within the intervention group, the effect over time on BW reduction was equal in participants with and without T2D, but in the control group, participants without T2D did not achieve significant weight change (Figure 2). Between the 6- and 12-month follow-ups, there was a significant weight reduction in the control group participants with T2D. All other weight changes at the 6- and 12-month follow-ups were not significant (Figure 2).

**Table 2:** Between-group differences in changes from baseline to the 6-month follow-up. Results in italics represent a significant change from baseline.

Characteristics			Intervention group at 6 months (n=126)		Control group at 6 months (n=71)		Between-group difference (95% CI)		*P* value
**Weight**									
	Change vs baseline (kg), mean (95% CI)	–*4.5* (–5.4 to –3.5)		–0.3 (–1.1 to 0.4)		4.2 (2.8 to 5.5)		<.001	
	Change vs baseline (%), mean (95% CI)	–*4.4* (–5.3 to –3.4)		–0.4 (–1.1 to 0.3)		3.9 (2.6 to 5.3)		<.001	
	Lost >5% bodyweight (n), %	49 (38.9)		6 (8.5)		30.4 (19.7 to 41.1)		<.001	
**Hemoglobin A_1c_**									
	Change (%), mean (95% CI)	–*0.5* (–0.6 to –0.3)		–*0.4* (–0.5 to –0.2)		0.1 (–0.2 to 0.4)		.49	
	Change (mmol/mol), mean (95% CI)	–*4.8* (–6.7 to –3.0)		–*3.8* (–5.9 to –1.8)		1.0 (–1.8 to 3.9)		.49	
	Reduction from >6.5% to <6.5% (only in T2D patients), n/N (%)^a^	22/63 (35)		9/34 (27)		8.4 (–10.4 to 27.3)		.39	
**Body composition**									
	BMI change (kg/m^2^), mean (95% CI)	–*1.5* (–1.8 to –1.2)		–0.1 (–0.4 to 0.1)		1.4 (0.9 to 1.8)		<.001	
	Change in hip circumference (cm), mean (95% CI)	–*5.5* (–6.5 to –4.6)		–*1.9* (–3.1 to –0.7)		3.6 (2.0 to 5.2)		<.001	
	Change in waist circumference (cm), mean (95% CI)	–*8.9* (–10.2 to –7.7)		–*3.3* (–4.8 to –1.8)		5.6 (3.6 to 7.6)		<.001	
	Change in waist/hip ratio (cm), mean (95% CI)	–*0.030* (–0.041 to –0.019)		–0.012 (–0.026 to 0.002)		0.018 (–0.000 to 0.036)		.052	
**Blood pressure**									
	Change in systolic pressure (mm Hg), mean (95% CI)	–1.4 (–3.6 to 0.8)		–0.3 (–3.4 to 2.9)		1.1 (–2.6 to 4.9)		.56	
	Change in diastolic pressure (mm Hg), mean (95% CI)	–*2.0* (–3.2 to –0.7)		–0.8 (–2.5 to 1.0)		1.2 (–0.9 to 3.3)		.27	
**Lipids**									
	Change in total cholesterol (mmol/ml), mean (95% CI)	–0.2 (–0.3 to 0.0)		0.1 (–0.1 to 0.3)		0.3 (–0.0 to 0.5)		.07	
	Change in high density lipoprotein (mmol/ml), median (95% CI)	–*0.1* (–0.2 to –0.0)		–0.1 (–0.1 to 0.0)		0.0 (–0.0 to 0.1)		.51	
	Change in triglyceride (mmol/ml), median (95% CI)	–*0.6* (–0.9 to –0.3)		0.7 (–1.6 to 3.1)		1.3 (–0.6 to 3.1)		.17	
	Change in low density lipoprotein (mmol/ml), median (95% CI)^a^	0.2 (–0.0 to 0.4)		*0.4* (0.1 to 0.6)		0.2 (–0.1 to 0.5)		.22	
Change in quality of life score, mean (95% CI)			0.0 (–0.0 to 0.0)		–0.0 (–0.0 to 0.0)		–0.0 (–0.0 to 0.0)		.14
Change in mental health score, mean (95% CI)			–0.3 (–0.9 to 0.3)		0.3 (–0.6 to 1.2)		0.6 (–0.5 to 1.6)		.27

^a^Calculated in 153/200 participants, including 95/127 in the intervention group and 59/73 in the control group.

**Table 3:** Between-group differences in changes from baseline to the 12-month follow-up. Results in italics represent a significant change from baseline.

Characteristics		Intervention group at 12 months (n=127)		Control group at 12 months (n=73)		Between-group difference, (95% CI)		*P* value
**Weight**								
	Change vs baseline (kg), mean (95% CI)	–*4.5* (–5.6 to –3.4)	–*1.5* (–2.7 to –0.2)		3.0 (1.3 to 4.8)		<.001	
	Change vs baseline (%), mean (95% CI)	–4.6 (–5.7 to –3.4)	–*1.4* (–2.6 to –0.1)		3.2 (1.4 to 5.0)		<.001	
	Lost >5% bodyweight (n), %	37.8 (48)	19.2 (14)		18.6 (6.2 to 30.9)		.01	
**Hemoglobin A_1c_**								
	Change (%), mean (95% CI)	–*0.5* (–0.7 to –0.4)	–*0.4* (–0.7 to –0.2)		0.1 (–0.2 to 0.4)		.41	
	Change (mmol/mol), mean (95% CI)	–*6.0* (–7.7 to –4.3)	–*4.9* (–7.4 to –2.4)		1.0 (–1.9 to 4.0)		.46	
	Reduction from >6.5% to <6.5% (only in in T2D patients), n/N (%)^a^	22/62 (36)	10/36 (28)		7.7 (–11.1 to 26.5)		.43	
**Body composition**								
	BMI change (kg/m^2^), mean (95% CI)	–*1.5* (–1.9 to –1.2)	–*0.5* (–0.9 to –0.1)		1.0 (0.4 to 1.7)		<.001	
	Change in hip circumference (cm), mean (95% CI)	–*5.9* (–7.0 to –4.8)	–*2.4* (–3.8 to –1.0)		3.5 (1.7 to 5.3)		<.001	
	Change in waist circumference (cm), mean (95% CI)	–*9.9* (–11.3 to –8.4)	–*4.5* (–6.6 to –2.5)		5.3 (2.8 to 7.8)		<.001	
	Change in waist/hip ratio (cm), mean (95% CI)	–*0.036* (–0.047 to –0.024)	–*0.019* (–0.036 to –0.002)		0.016 (0.003 to 0.0361)		.11	
**Blood pressure**								
	Change in systolic pressure (mm Hg), mean (95% CI)	–*3.3* (–5.3 to –1.4)	–*4.7* (–8.0 to –1.3)		–1.3 (–5.0 to 2.3)		.47	
	Change in diastolic pressure (mm Hg), mean (95% CI)	–*2.4* (–3.6 to –1.2)	–1.4 (–3.7 to 0.9)		1.0 (–1.4 to 3.4)		.40	
**Lipids**								
	Change in total cholesterol (mmol/ml), mean (95% CI)	–*0.4* (–0.5 to –0.2)	–*0.2* (–0.5 to –0.0)		0.1 (–0.2 to 0.4)		.42	
	Change in high density lipoprotein, (mmol/ml), median, mean (95% CI)	0.6 (–1.0 to 2.2)	–*0.2* (–0.3 to –0.2)		–0.8 (–2.9 to 1.3)		.44	
	Change in triglycerides (mmol/ml), median, (95% CI)	–*0.8* (–1.1 to –0.6)	–*0.8* (–1.1 to –0.5)		0.05 (–0.4 to 0.5)		.81	
	Change in low density lipoprotein (mmol/ml), median, (95% CI)^a^	0.2 (0.0 to 0.3)	0.3 (0.0 to 0.5)		0.1 (–0.2 to 0.4)		.58	
Change in quality of life score, mean (95% CI)		0.0 (–0.0 to 0.0)		–0.0 (–0.0 to 0.0)		–0.0 (–0.0 to 0.0)		.47
Change in mental health score, mean (95% CI)		0.4 (–0.2 to 1.0)		0.3 (–0.6 to 1.2)		–0.1 (–1.1 to 0.9)		.84

^a^Calculated in 153/200 participants, including 95/127 in the intervention group and 59/73 in the control group.

### Secondary Outcome

At the 6-month follow-up, our secondary outcome, HbA_1c_, was equally reduced in both groups. At the 12-month follow-up, HbA_1c_ in both the intervention group (–0.5%, 95% CI –0.7 to –0.4) and the control group (–0.4%, 95% CI –0.7 to –0.2) were still equally reduced (Table 3). The largest reduction of HbA_1c_ in the intervention group was seen within the first 6 months (Figure 2). Although Figure 2 might seem to indicate that the intervention only reduced HbA_1c_ in participants with T2D, T2D did not interact with the effect of the intervention on HbA_1c_ (all values: *P*>.43). The reduction in HbA_1c_ at the 12-month follow-up among participants in the intervention group with T2D was greater (–0.7%, 95% CI –1.1 to –0.4) than the reduction in participants without T2D (–0.4%, 95% CI –0.4 to –0.3). From the 6-month follow-up to the 12-month follow-up, participants without T2D significantly reduced HbA_1c_, but there were no significant changes within the group of participants with T2D (Figure 2). The proportion of participants in the intervention group whose HbA_1c_ became normal was significantly greater at the 12-month follow-up (54/27, 43%) than at baseline (33/127, 25.9%), but HbA_1c_ becoming normal was not significantly more prevalent than in the control group (Multimedia Appendix 3).

### Tertiary Outcomes

At the 6- and 12-month follow-ups, mean BMI decreased significantly in both groups, but significantly more so in the intervention group (–1.5 kg/m^2^, 95% CI –1.9 to –1.2 vs –0.5 kg/m^2^, 95% CI –0.9 to –0.1; *P*<.001). The waist/hip ratio was reduced significantly in both groups, but there was no significant between-group difference (Table 3).

At the 12-month follow-up, blood pressure, total cholesterol, and TG were reduced in both groups without any between-group differences (Table 3). HDL-C was decreased in both groups at the 6-month follow-up. At the 12-month follow-up, HDL-C was still decreased in the control group but was nonsignificantly increased in the intervention group. There were no statistically significant between-group differences at either the 6- or 12-month follow-ups.

At both the 6- and 12-month follow-ups, quality of life and mental health were unchanged in both groups (Tables 2 and 3). In general, medications (assessed as the defined daily dose for glucose-lowering and blood pressure–lowering drugs) did not change in any of the groups. However, use of dipeptidyl peptidase-4 inhibitors (DPP4s) decreased significantly in the intervention group, while use increased in the control group (*P*=.03) (Multimedia Appendix 3). Use of angiotensin-converting enzyme inhibitors increased insignificantly in the intervention group and decreased, although not significantly, in the control group (*P*=.06) (Multimedia Appendix 3).

## Discussion

### Principal Results

The main objective of this 12-month RCT was to see if individualized digital lifestyle coaching, enabled by an eHealth tool, could help people living with obesity with or without T2D to achieve and maintain a significant weight loss. This objective was met, with a mean weight loss of 4.5 kg in the intervention group, compared to 1.5 kg in the control group, after 12 months of follow-up.

### Comparison With Prior Work

These findings support previous studies that used eHealth solutions to promote lifestyle changes [24-26]. Our results are in line with a recent meta-analysis of studies using in-person behavioral counseling together with an eHealth intervention that showed a BW reduction of –4.65% [25].

The beneficial effect of the intervention in our study was probably due to the combination of face-to-face coaching and asynchronous eHealth with a different BCT, which has been proven effective in other studies [27,28]. The initial establishment of an honest and trustworthy relationship was found to be relevant in the qualitative interviews that the research team conducted while developing this study’s eHealth tool [13-15]. This finding is supported by smaller studies demonstrating that patients who found lifestyle changes challenging appeared to improve health behaviors when they used digital coaching that built on an empathetic relationship [16]. Observational retrospective studies suggest digital eHealth intervention incorporating personal coaching and BCTs may promote weight loss better over a 12-month period compared to studies with either face-to-face coaching or eHealth alone [29,30]. However, RCTs of eHealth solutions providing individualized coaching with follow-up at 12 months are sparse [31,32].

From baseline to the 12-month follow-up, HbA_1c_ was reduced in both the intervention and control groups without a significant difference between the groups. This lack of difference was in contrast to a prior meta-analysis [26] and could not be explained by the small decrease in DPP4 use in the intervention group. The fact that the intervention did not reduce HbA_1c_ significantly more in participants with T2D may reflect blood glucose already being well regulated at baseline in most of the participants with T2D (mean HbA_1c_ was 6.6%). It is important that HbA_1c_ was significantly reduced between the 6- and 12-month follow-ups in the participants without T2D in both groups, although BW was not reduced in the control group without T2D, suggesting that lifestyle changes other than weight reduction, such as more exercise, may have contributed to the HbA_1c_ reduction.

Systolic blood pressure was significantly reduced in both groups at the 12-month follow-up, without a significant between-group difference, which might reflect the blood pressure reduction being a consequence of participation in the study (ie, the “healthy participator” effect) and only partly secondary to the weight loss. The same explanation is likely for the lipid findings. The lack of change in quality of life and mental health in both groups probably reflects these questionnaires being rather broad and therefore very robust toward changes in selective interventions. It is not unlikely that a specific overweight questionnaire would have picked up improvements related to the observed weight loss, but there would probably not have been a significant difference between the groups.

### Limitations

The dropout rate at the 12-month follow-up was 138 of 338 (40.8%), which is similar to attrition rates reported in other studies [9]. Although this could have created attrition bias, the participants who dropped out were closely comparable to the participants who came to the 12-month follow-up, and the characteristics of the participants in the intervention and control groups who came to the 12-month follow-up did not differ from each other at baseline. This may reflect many of the dropouts occurring at random due to COVID-19 restrictions. However, the relatively high number of dropouts reduced the power, making a subgroup analysis of participants with T2D difficult, with a high risk of false negative results, and may explain the nonsignificant effect of the intervention on HbA_1c_. Another limitation of this study was the number of participants who came to the 6-month follow-up but missed the 12-month follow-up, possibly due to COVID-19 restrictions. As our clinical end points needed physical attendance, it was not possible to follow up with participants who dropped out. However, we repeated the analysis with imputation used to replace missing values. For 3 participants, the 12-month data was used to impute missing 6-month data, and for 35 participants, the 6-month data was used to impute missing 12-month data. This analysis obtained similar results. All the participants randomized to the intervention group who stayed in the study used the eHealth tool, indicating that if the eHealth tool is used in the future, the therapist will quickly be able to identify who is not satisfied with the individualized digital coaching. For these subjects, it will be possible for the therapist to recommend other treatment strategies.

Although evidence suggests that human feedback and coaching is an important element for success, our study design did not allow us to comment on the relative effectiveness of the components of this study’s eHealth approach. On the other hand, the randomized design of our study is a strength, showing that together with the relatively low cost of the intervention, a large scale-up seems possible.

### Conclusion

It is possible to induce and maintain lifestyle changes leading to significant and sustainable 12-month, long-term weight loss among people living with obesity with or without T2D using individualized digital lifestyle coaching, in comparison to usual care. These findings suggest that coaching with an eHealth tool based on real-time monitoring incorporating personal coaching and BCTs through smartphones can lead to improved lifestyles that may have the potential to further reduce the incidence and severity of NCDs.

## Appendix

Multimedia Appendix 1 Template of the Intervention Description and Replication checklist for the LIVA 2.0 program.

Multimedia Appendix 2 YouTube video showing an example of the Liva app used in real life.

Multimedia Appendix 3 Supplementary tables and figures.

Multimedia Appendix 4 CONSORT-eHEALTH checklist (V 1.6.1).

## Abbreviations

BCT: behavior change technique
BW: body weight
DPP4: dipeptidyl peptidase-4 inhibitor
eHealth: electronic health
EQ-5D-5L: European Quality of Life-5 Dimensions
GP: general practitioner
HbA_1c_: hemoglobin A_1c_
HCP: health care professional
HDL-C: high-density lipoprotein cholesterol
LDL-C: low-density lipoprotein cholesterol
mHealth: mobile health
NCD: noncommunicable disease
RCT: randomized controlled trial
SMART: specific, measurable, attainable, relevant, timely
SWEMWBS: Short-Warwick-Edinburgh Mental Well-being Scale
T2D: type 2 diabetes
TG: triglyceride
