# Supplementary material for: Long-term Weight Loss in a Primary Care–Anchored eHealth Lifestyle Coaching Program: Randomized Controlled Trial
Source: J Med Internet Res. 2022 Sep 23;24(9):e39741. doi: 10.2196/39741 (PMC9547330; doi:10.2196/39741)
Supplement: Multimedia Appendix 1 [file jmir_v24i9e39741_app1.docx]

Multimedia appendix 1
Template of the Intervention Description and Replication checklist for the LIVA 2.0 program.

| Item | Description |
| --- | --- |
| eHealth coaching sessions | Prior to the intervention, the health coaches receive training in setting SMART (specific, measurable, agreed upon, realistic, and time-based) goals with the participants using the eHealth solution LIVA 2.0 and in setting up action and coping plans that address barrier identification and problem solving. Participants in the intervention group have one or two personal meetings (face-to-face or digital) with their health coach, followed by asynchronous web-based consultations based on dialog by means of text or video. The consultations address the participant’s registrations, goal setting, and questions regarding diet, exercise, and lifestyle plans, taking into consideration chronic diseases. The LIVA 2.0 app is set up with short explanations about different functions and notifications and reminders to the participants to register and give feedback about the health coaching. The sessions provide the participant with information in relation to their status, specific focus on goals, and recommendations on how to improve their behaviors.  Include BCT^a^ from CALO-RE^b^ taxonomy hereafter referred to as BCT) as follows: provide information on the consequences of the behavior in *general* and *to the* *individual*, goal setting, behavior and outcome, action planning, and barrier identification/problem solving; set graded tasks; prompt review of behavioral goals; prompt review of outcome goals; prompt rewards contingent on effort or progress toward behavior; prompt generalization of a target behavior; and provide feedback on performance. |
| Goals and inputs | Goals and inputs are always driven by the participant and are available to the participant, who can choose the focus area, set specific goals, and keep record of specified behaviors by reporting them on a daily, weekly, or monthly basis. This allows the user and the health coach to follow progress or setbacks as the numbers and registrations are visualized using graphs and curves. All coaching by the health coach follows national guidelines from the Danish National Board of Health. |
| Dietary goals and plans | Dietary goals and plans can be set at many different levels (eg, from simple changes aiming at changing one meal a day to more complex changes aiming at a completely new diet for the remedying of digestion problems). |
| Physical activity goals and plans | Physical activity goals and plans involve goal setting and recording of the type of physical activity and time for executing the given physical activity. The participant receives advice and/or a video on activities in a variety of contexts to foster physical activity as a more integrated part of life (BCT: provide instruction on how to perform the behavior, prompt generalization of a target behavior, and provide relapse prevention/coping planning). |
| Life goals | Goals on a healthy joyful life as the participant sees it (e.g., daily life with less stress, stronger social bonds with friends and family, coping skills for diseases, etc.). |
| Weight | Set the current weight and goal for a lower or higher weight and register new measurements on a daily, weekly, or monthly basis. |
| Steps | When downloading the LIVA 2.0 app, the participant can accept direct import of their information on steps recorded on a smartphone, and tailored messages on progress toward a set goal appear simultaneously (BCT: teach-to-use prompts/cues). Step count monitoring is encouraged but not required to enter the LIVA study. Some participants will have other ways of registering their physical activity level. |
| Pain, sleep, and mood | Give daily feedback on pain, sleep, and mood, which can affect the ability to perform a given behavior (BCT: relapse prevention/coping planning). |
| Smoking | Set goals to bring down the number of cigarettes smoked on a daily basis, leading to cessation. |
| Blood glucose and blood pressure | Keep a record of specified measures expected to be influenced by the different behavior changes addressed. In LIVA, this includes blood glucose and blood pressure measurements (BCT: prompt self-monitoring of behavioral outcome and provide information on consequences of the behavior in *general* and *for the* *individual*). |
| Forum | Online forum where the participant can exchange knowledge, gain social support, and build new relationships, and the health coach can provide advice to the participant (BCT: plan social support/change). |
| Coaching providers | Health coaches with basic training as nurses, physiotherapists, dieticians, or occupational therapists. In Denmark, all four education types consist of 420 European Credit Transfer System (ECTS) Points (3.5 years of full-time education). In addition to their education as health care professionals, they all undergo special training in using digital health coaching and practice digital health coaching for at least 2 years. |
| Coaching approach | Individually delivered via the app or web-based delivery. |
| Coaching location | Initial personal meetings in municipality health centers, general practice medical centers, or the research unit for general practice at the University of Southern Denmark or over the internet, and then, solely web-based delivery. |
| Coaching time and quantity | The initial consultation with a health coach is estimated to last for approximately 45-60 minutes. The subsequent asynchronous eHealth coaching sessions are carried out once a week in the first 6 months and then every month for the last 6 months for maintenance. Thereafter, the participant could receive two eHealth coaching sessions and use LIVA 2.0 as a personal BCT tool (BCT: use of follow up prompts). |
| Tailoring | Every participant receives personalized eHealth coaching sessions from their designated health coach. The provided feedback is based on the participant’s inputs on LIVA 2.0. |

^a^BCT: behavior change technique.

^b^CALO-RE: Coventry, Aberdeen and LOndon-REfined taxonomy.
