# Supplementary material for: Long-term Weight Loss in a Primary Care–Anchored eHealth Lifestyle Coaching Program: Randomized Controlled Trial
Source: J Med Internet Res. 2022 Sep 23;24(9):e39741. doi: 10.2196/39741 (PMC9547330; doi:10.2196/39741)
Supplement: Multimedia Appendix 2 [file jmir_v24i9e39741_app2.docx]

**SUPPLEMENTARY FILE:**

**youtube video showing an example of the LIVA app used in real life, as requested by one of the reviewers**

<https://www.youtube.com/watch?v=-dlpN1znH7U&feature=emb_logo>
