# Supplementary material for: Long-term Weight Loss in a Primary Care–Anchored eHealth Lifestyle Coaching Program: Randomized Controlled Trial
Source: J Med Internet Res. 2022 Sep 23;24(9):e39741. doi: 10.2196/39741 (PMC9547330; doi:10.2196/39741)
Supplement: Multimedia Appendix 3 [file jmir_v24i9e39741_app3.docx]

**Multimedia Appendix 3. Supplementary tables and figures**

**Table S1.** Baseline characteristics

|  | Participants who discontinued after baseline | Participants coming to 12 months  follow-up | Total | *P* value |
| --- | --- | --- | --- | --- |
|  | (n=138) | (n=200) | (n=338) |  |
| **Age, mean (sd)** | 51.9 (11.2) | 52.3 (10.8) | 52.2 (11.0) | .75 |
| **Sex, n (%)** |  |  |  |  |
| Female | 86 (62.3) | 127 (63.5) | 213 (63.0) |  |
| Male | 52 (37.7) | 73 (36.5) | 125 (37.0) | .83 |
| **Diabetes, n (%)** |  |  |  |  |
| Yes | 70 (50.7) | 98 (49.0) | 168 (49.7) |  |
| No | 68 (49.3) | 102 (51.0) | 170 (50.3) | .76 |
| **Education, n (%)** |  |  |  |  |
| Don't know | 4 (2.9) | 2 (1.0) | 6 (1.8) |  |
| None | 21 (15.3) | 34 (17.0) | 55 (16.3) |  |
| Short | 32 (23.4) | 52 (26.0) | 84 (24.9) |  |
| Middle | 68 (49.6) | 91 (45.5) | 159 (47.2) |  |
| Long | 12 (8.8) | 21 (10.5) | 33 (9.8) | .63 |
| **Marital status, n (%)** |  |  |  |  |
| Married | 75 (54.3) | 141 (70.5) | 216 (63.9) |  |
| Unmarried^a^ | 61 (44.2) | 56 (28.0) | 117 (34.6) |  |
| Widdow | 2 (1.4) | 3 (1.5) | 5 (1.5) | .01 |
| **Occupational status, n (%)** |  |  |  |  |
| Employed | 87 (63.5) | 144 (72.0) | 231 (68.5) |  |
| Out of work^b^ | 20 (14.6) | 16 (8.0) | 36 (10.7) |  |
| Retired | 24 (17.5) | 37 (18.5) | 61 (18.1) |  |
| Student | 6 (4.4) | 3 (1.5) | 9 (2.7) | .08 |
| **Body composition** |  |  |  |  |
| Weight (kg), mean (sd) | 104.4 (15.7) | 103.7 (15.7) | 104.0 (15.7) | .69 |
| BMI (kg/m2), mean (sd) | 35.7 (4.6) | 35.3 (3.8) | 35.5 (4.1) | .30 |
| Hip (cm), mean (sd) | 122.9 (12.1) | 121.3 (9.8) | 122.0 (10.8) | .18 |
| Waist (cm), mean (sd) | 119.0 (12.1) | 119.0 (11.6) | 119.0 (11.8) | .99 |
| WHR (cm), mean (sd) | 0.971 (0.085) | 0.983 (0.089) | 0.978 (0.087) | .22 |
| **Glycemic control** |  |  |  |  |
| HbA_1c_ (%), mean (sd) | 6.6 (1.2) | 6.6 (1.3) | 6.6 (1.2) | .90 |
| HbA_1c_ (mmol), mean (sd) | 47.8 (13.1) | 48.3 (13.7) | 48.1 (13.4) | .75 |
| HbA_1c_ <6.5%, n (%) | 80 (58.0) | 111 (55.5) | 191 (56.5) | .65 |
| **Blood pressure** |  |  |  |  |
| Systolic (mmHg), mean (sd) | 134.3 (18.7) | 130.9 (14.8) | 132.3 (16.6) | .07 |
| Diastolic (mmHg), mean (sd) | 89.4 (9.8) | 86.2 (9.0) | 87.5 (9.5) | .01 |
| **Lipids** |  |  |  |  |
| Totalcholesterol (mmol/l), mean (sd) | 4.9 (1.2) | 4.9 (1.2) | 4.9 (1.2) | .79 |
| HDL(mmol/l), median (iqr) | 1.2 (0.7) | 1.2 (0.5) | 1.2 (0.6) | .77 |
| LDL(mmol/l), median (iqr) | 2.3 (1.6) | 2.2 (1.5) | 2.3 (1.5) | .71 |
| Triglycerid (mmol/l), median (iqr) | 3.0 (1.9) | 2.6 (2.3) | 2.8 (2.2) | .38 |
| **Mental health score, mean (sd)** | 24.2 (3.9) | 24.8 (3.5) | 24.5 (3.7) | .14 |
| **Quality of life score, mean (sd)** | 0.7 (0.1) | 0.8 (0.1) | 0.8 (0.1) | .02 |

^a^Single or divorced, ^b^Maternary leave, unemployment benefit, or cash benefit, ^c^The Short-Warwick-Edinburgh Mental Well-being Scale (SWEMWBS). Index ranges from 7-35, ^d^An index calculated based on the different dimensions measured in the European Quality of life - 5 Dimensions scheme - EQ-5D-5L. Index ranges from 0.35 to 1.0.

**Table S2.** Baseline medicine

|  | Participants who discontinued after baseline | Participants completing 12 months  intervention | Total | *P* value |
| --- | --- | --- | --- | --- |
|  | (n=138) | (n=200) | (n=338) |  |
| **Glucose lowering** |  |  |  |  |
| Metformin, n (%) | 43 (31.2) | 87 (44.2) | 130 (38.8) | .02 |
| GLP1, n (%) | 7 (5.1) | 16 (8.1) | 23 (6.9) | .28 |
| Insulin, n (%) | 4 (2.9) | 16 (8.1) | 20 (6.0) | .051 |
| SGLT2, n (%) | 3 (2.2) | 15 (7.6) | 18 (5.4) | .03 |
| DPP4, n (%) | 3 (2.2) | 7 (3.6) | 10 (3.0) | .47 |
| Solfonylurea, n (%) | 1 (0.7) | 2 (1.0) | 3 (0.9) | .78 |
| **Blood pressure lowering** |  |  |  |  |
| ARB/ACE, n (%) | 28 (20.3) | 57 (28.5) | 84 (25.1) | .09 |
| Calcium antagonist, n (%) | 7 (5.1) | 23 (11.7) | 30 (9.0) | .04 |
| Diuretics, n (%) | 7 (5.1) | 18 (9.1) | 25 (7.5) | .16 |
| Beta blocker, n (%) | 3 (2.2) | 11 (5.6) | 14 (4.2) | .12 |
| Alpha blocker, n (%) | 1 (0.7) | 0 (0.0) | 1 (0.3) | .23 |
| Aldosteron antagonist, n (%) | 2 (1.4) | 2 (1.0) | 4 (1.2) | .72 |
| **Lipid lowering, n (%)** | 34 (24.6) | 67 (34.0) | 101 (30.1) | .07 |

**Table S3.** Medicine changes in DDD from baseline to 12 months

|  | Intervention group completing 12 months | Control group completing 12 months | Total | *P* value |
| --- | --- | --- | --- | --- |
|  | (n = 127) | (n=73) | (n=200) |  |
| Glucose lowering |  |  |  |  |
| Overall change, mean (sd) | -0.02 (0.45) | -0.01 (0.36) | -0.01(0.42) | .89 |
| Metformin DDD change, mean (sd) | -0.01 (0.17) | -0.04 (0.20) | -0.02 (0.18) | .20 |
| SGLT2 DDD change, mean (sd) | 0.01 (0.14) | 0.03 (0.22) | 0.02 (0.17) | .39 |
| Insulin DDD change, mean (sd) | -0.04 (0.25) | -0.01 (0.11) | -0.03 (0.21) | .43 |
| GLP1 DDD change, mean (sd) | 0.03 (0.23) | -0.01 (0.13) | 0.01 (0.20) | .19 |
| DPP4 DDD change, mean (sd) | -0.02 (0.15) | 0.03 (0.16) | -0.01 (0.16) | .03 |
| Solfonylurea DDD change, mean (sd) | 0.02 (0.18) | 0.00 (0.00) | 0.01 (0.14) | .45 |
| Blood pressure lowering |  |  |  |  |
| Overall change, mean (sd) | -0.05 (0.46) | -0.24 (1.13) | -0.12 (0.78) | .11 |
| ARB+ACE DDD change, mean (sd) | -0.01 (0.24) | -0.17 (0.93) | -0.07 (0.59) | .06 |
| Calcium antagonist DDD change, mean (sd) | -0.02 (0.32) | -0.02 (0.18) | -0.02 (0.28) | .94 |
| Diuretics DDD change, mean (sd) | -0.02 (0.12) | -0.04 (0.20) | -0.03 (0.16) | .45 |
| Beta blocker DDD change, mean (sd) | 0.00 (0.00) | -0.00 (0.04) | -0.00 (0.02) | .19 |

**Figure S1.** HbA_1c_ changes in the intervention group from baseline, 6 months, 12 months (n=127)

**Figure S2.** HbA_1c_ changes in the control group from baseline, 6 months, 12 months (n=73)
